# ROR2 receptor promotes the migration of osteosarcoma cells in response to Wnt5a

**DOI:** 10.1186/s12935-017-0482-y

**Published:** 2017-11-29

**Authors:** Bin Dai, Ting Yan, Ailiang Zhang

**Affiliations:** 1 Department of Orthopedics, Binhai County People’s Hospital, Binhai, 224500 Jiangsu China; 2 Safety Assessment and Research Center for Drug, Pesticide and Veterinary Drug of Jiangsu Province, School of Public Health, Nanjing Medical University, Nanjing, 211166 Jiangsu China; 3 Spine Surgery, Third Affiliated Hospital of Soochow University, Changzhou, 213003 Jiangsu China

**Keywords:** ROR2, Wnt5a, Osteosarcoma, Migration

## Abstract

**Background:**

We have reported that the phosphatidylinositol-3 kinase (PI3K)/Akt/RhoA signaling pathway mediates Wnt5a-induced cell migration of osteosarcoma cells. However, the specific receptors responding to Wnt5a ligand remain poorly defined in osteosarcoma metastasis.

**Methods:**

Wound healing assays were used to measure the migration rate of osteosarcoma cells transfected with shRNA or siRNA specific against ROR2 or indicated constructs. We evaluated the RhoA activation in osteosarcoma MG-63 and U2OS cells with RhoA activation assay. A panel of inhibitors of PI3K and Akt treated osteosarcoma cells and blocked kinase activity. Western blotting assays were employed to measure the expression and activation of Akt. Clonogenic assays were used to measure the cell proliferation of ROR2-knockdown or ROR2-overexpressed osteosarcoma cells.

**Results:**

Wnt5a-induced osteosarcoma cell migration was largely abolished by shRNA or siRNA specific against ROR2. Overexpression of RhoA-CA (GFP-RhoA-V14) was able to rescue the Wnt5a-induced cell migration blocked by ROR2 knockdown. The Wnt5a-induced activation of RhoA was mostly blocked by ROR2 knockdown, and elevated by ROR2 overexpression, respectively. Furthermore, we found that Wnt5a-induced cell migration was significantly retarded by RhoA-siRNA transfection or pretreatment of HS-173 (PI3Kα inhibitor), MK-2206 (Akt inhibitor), A-674563 (Akt1 inhibitor), or CCT128930 (Akt2 inhibitor). The activation of Akt was upregulated or downregulated by transfected with ROR2-Flag or ROR2-siRNA, respectively. Lastly, Wnt5a/ROR2 signaling does not alter the cell proliferation of MG-63 osteosarcoma cells.

**Conclusions:**

Taken together, we demonstrate that ROR2 receptor responding to Wnt5a ligand activates PI3K/Akt/RhoA signaling and promotes the migration of osteosarcoma cells.

## Background

Wnt5a, a non-transforming Wnt family member, plays complicated roles in oncogenesis and cancer metastasis, exerting both oncogenic and tumor suppressive effects [1]. Wnt5a functions as a promoter in osteosarcoma progression [2–4]. Our previous study illuminates that Wnt5a mediates the migration of osteosarcoma cells via elevating the phosphatidylinositol-3 kinase (PI3K)/Akt and RhoA signaling [5, 6]. However, there is still unknown which receptors responds to Wnt5a signaling and participates in osteosarcoma metastasis.

Wnt factors can bind to three types of receptors, which are identified as frizzled family receptors (Fzd), low-density lipoprotein receptor-related protein (LRP), and receptor tyrosine kinase-like orphan receptor (ROR) [7–9]. Wnt5a competes with Wnt3a for binding to Fzd2 and thereby inhibits Wnt3a-dependent LRP6 phosphorylation and β-catenin-dependent Wnt signaling [10]. Wnt5a also can activate β-catenin-dependent pathway and induce secondary axis formation in *Xenopus* embryos that express the Fzd5 receptor [11, 12]. Wnt5a induces heterooligermization of ROR1/ROR2, which activates RhoA and Rac1 and then enhances leukemia-cell chemotaxis and proliferation [13]. Purified Wnt5a protein inhibits canonical Wnt/β-catenin signaling in ROR2-expressed cells, but also induces canonical Wnt/β-catenin signaling in cells that expressed Fzd4 and LRP5 [14].

Although there are substantial evidences given that Wnt5a binds to diverse receptors and promotes cellular behaviors (e.g. cell chemotaxis, cell proliferation), it is still much uncertainty regarding the receptor responds to Wnt5a and regulates metastatic behavior of osteosarcoma cells. Here, we demonstrates that ROR2 receptor activates PI3K/Akt/RhoA signaling and mediates Wnt5a-induced the migration of osteosarcoma cells.

## Methods

### Cell culture

Human MG-63 and U2OS osteosarcoma cell lines were purchased from Cells Resource Center of Shanghai Institutes for Biological Sciences, Chinese Academy of Sciences (Shanghai, China). These cells were cultured in Dulbecco-modified Eagle’s medium (DMEM) supplemented with 10% fetal bovine serum (FBS, Hyclone, Logan, UT), at 37 °C in a humidified atmosphere with 5% CO_2_. MG-63 and U2OS cells were plated onto 6-well cell culture clusters (Costar) and grown to 80% confluence, and then serum-starved for 24 h. These cells were subsequently treated with HS-173 (PI3Kα inhibitor), MK-2206 (Akt inhibitor), A-674563 (Akt1 inhibitor), or CCT128930 (Akt2 inhibitor) (Selleck, Houston, TX) before RhoA activation assays and wound healing assays.

### Plasmids, small interfering RNA (siRNA) and short hairpin RNA (shRNA)

The construct ROR2-Flag was purchased from Addgene (Cambridge, MA). The constructs GFP-RhoA-N19, GFP-RhoA-V14 and vectors were kindly provided by Dr. Zhu (Nanjing Medical University, China). SiRNA duplexes specific for ROR2 or RhoA (Santa Cruz Biotechnology, Santa Cruz, CA) were transiently transfected into MG-63 and U2OS cells by using Lipofectamine 2000 reagent (Invitrogen, Carlsbad, CA) in serum-free OPTI-MEM according to the manufacturer’s instructions. The commercial siRNAs are a pool of 3 siRNAs specifically targeting ROR2 or RhoA, respectively (Santa Cruz Biotechnology). The cells were switched to fresh medium containing 10% FBS 6 h after the transfection and cultured for 48 h. The cells transfected with indicated constructs or siRNAs were used for analyzing the protein expression and cell migration.

ROR2 shRNA Plasmid (Santa Cruz Biotechnology) is a pool of three target-specific lentiviral vector plasmids each encoding 19–25 nt (plus hairpin) shRNAs designed to knock down gene expression. Each plasmid contains a puromycin resistance gene for the selection of cells stably expressing shRNA. ShRNAs specific for ROR2 or scrambled shRNAs were transfected into MG-63 cells using Lipofectamine 2000 reagent (Invitrogen). The cells were switched to fresh medium containing 10% FBS 6 h after the transfection and cultured for 48 h. After selection with puromycin (1 μg/mL) and serial limit dilutions, the ROR2 expression was controlled by Western blotting assays. Four selected clones of control and positive cells were pooled in order to avoid clonal variation. All cells were maintained in a 37 °C incubator with 5% CO_2_ and cultured as the parental cells.

### Wound healing assay

MG-63 and U2OS cells transfected with indicated constructs or siRNA and stable ROR2 knockdown MG-63 cells were plated onto 96-well cell culture clusters (Costar) and grown to confluence, and then serum starved for 24 h. The monolayer cells were scratched manually with a plastic pipette tip, and after two washes with PBS, the wounded cellular monolayer was allowed to heal for 12 h in DMEM containing 100 ng/mL recombinant Wnt5a (rWnt5a) (R&D Systems). Photographs of central wound edges per condition were taken at time 0 and 12 h after scratched using digital camera (Nikon, Tokyo, Japan).

### RhoA activation assay (G-LISA small GTPase activation assays)

MG-63 and U2OS cells transfected with ROR2-Flag or ROR2-siRNA and stable ROR2 knockdown MG-63 cells were seeded into 6-well plates and treated with 100 ng/mL Wnt5a. The experiments were then performed according to the manufacturer’s protocol (Cytoskeleton Inc., Denver, CO) [15]. G-LISA small GTPase activation assays offer a fast and sensitive method for performing small G-protein activation assays. Briefly, equal protein concentration in all samples is a prerequisite for accurate comparison between samples in GTPase activation assays. Cell extracts were equalized with ice-cold Lysis Buffer containing protease inhibitor cocktail to give identical protein concentrations. The Precision Red Advanced Protein Assay Reagent is a simple one step procedure that results in a red to purple/blue color change characterized by an increase in absorbance at 600 nm. Add 10 µL of each lysate or Lysis Buffer into the well of a 96 well plate. Add 290 µL of Precision Red Advanced Protein Assay Reagent to each well. Incubate for 1 min at room temperature. Blank spectrophotometer with 290 µL of Precision Red plus 10 µL of lysis buffer at 600 nm. Read absorbance of lysate samples. The activation of RhoA was normalized to the control group. RhoA activation assays were performed in triplicate.

### Western blotting

Subconfluent cells were washed twice with PBS, and then lysed with ice-cold RIPA lysis buffer (50 mmol/L Tris, 150 mmol/L NaCl, 1% Triton X-100, 1% sodium deoxycholate, 0.1% SDS, 1 mmol/L sodium orthovanadate, 1 mmol/L sodium fluoride, 1 mmol/L EDTA, 1 mmol/L PMSF, and 1% cocktail of protease inhibitors) (pH7.4). The lysates were then clarified by centrifugating at 12,000*g* for 20 min at 4 °C. The protein extracts were separated by SDS-PAGE. The immunoblotting procedure was performed as described [16] and the following antibodies were used: rabbit anti-ROR2 antibody, mouse anti-GAPDH antibody (Proteintech, Wuhan, China), rabbit anti-Akt antibody, rabbit anti-phospho-Akt (p-Ser473) antibody (Cell Signaling Technology, Danvers, MA). Protein bands were detected by incubating with horseradish peroxidase-conjugated antibodies (Santa Cruz Biotechnology) and visualized with ECL reagent (Thermo Scientific, Rockford, IL). The gray values were taken by Tanon imaging analysis system (Tanon, Shanghai, China).

### Clonogenic assay

MG-63 cells transfected with ROR2-Flag or ROR2-siRNA and stable ROR2 knockdown MG-63 cells were placed into 6-well plates (5000 cells/well), incubated with 100 ng/mL Wnt5a at 37 °C for 2 weeks, fixed and stained with crystal violet. The mean ± SD number of colonies was counted under a microscope from three independent replicates.

### Statistical analysis

All experiments here were repeated at least three times, with independent treatments, each of which showed essentially the same results. The data were analyzed using Student’s *t* test by SPSS statistical software package. All the results were expressed as mean ± SD. For all analyses a two-sided *p* < 0.05 was deemed statistically significant.

## Results

### ROR2 participates in Wnt5a-induced osteosarcoma cell migration

**Fig. 1:**
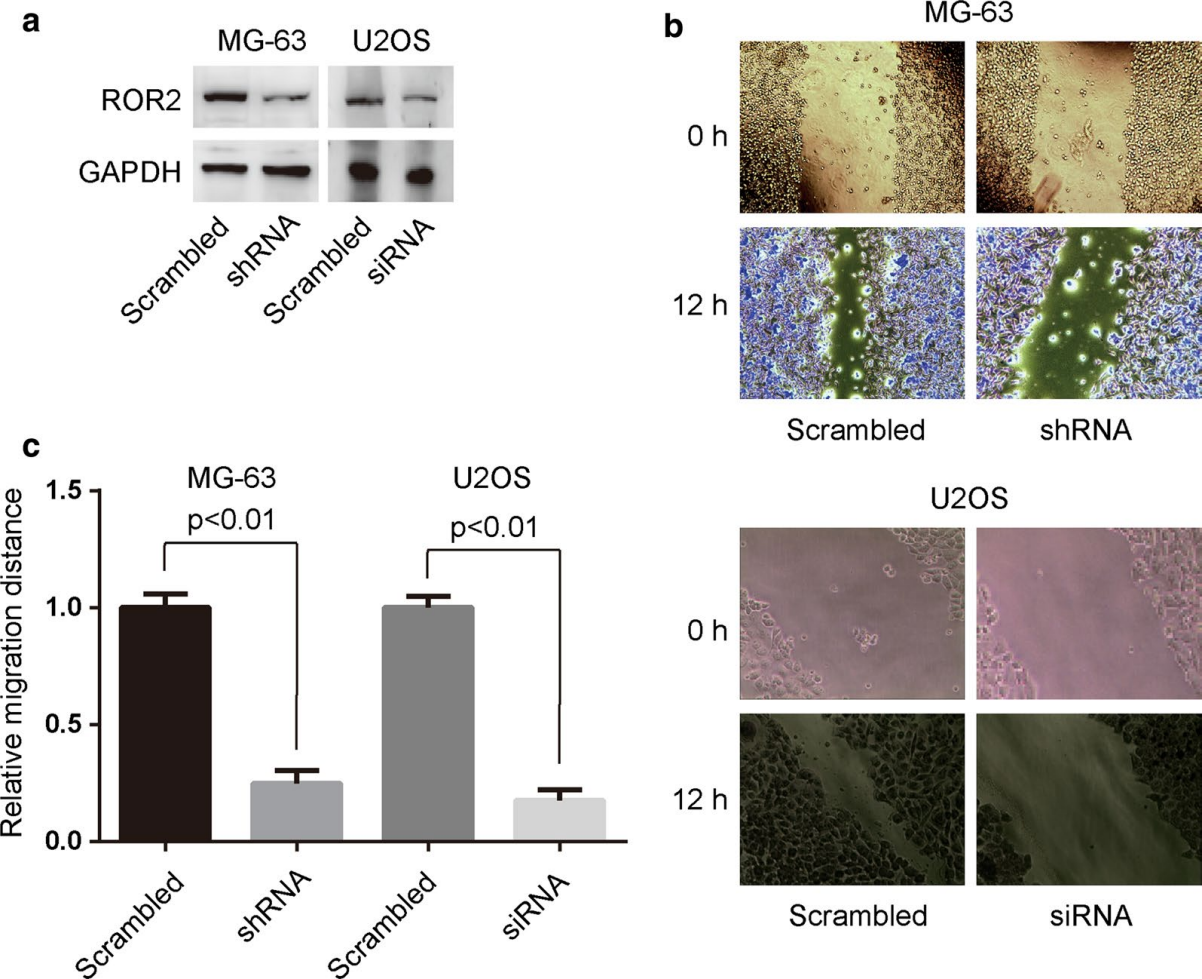
ROR2 participates in Wnt5a-induced osteosarcoma cell migration. **a** Stable ROR2 knockdown MG-63 cells and osteosarcoma U2OS cells transfected with ROR2-siRNA or scrambled siRNA were subjected to Western blotting assays. The expression of ROR2 in MG-63 and U2OS cells was significantly knockdown by specific shRNAs or siRNAs targeting ROR2. GAPDH was used as an internal control. **b** and **c** Stable ROR2 knockdown MG-63 cells and osteosarcoma U2OS cells transfected with ROR2-siRNA or scrambled siRNA were subjected to wound healing assays. Cells incubated with 100 ng/mL Wnt5a were allowed to migrate for 12 h. Data were presented as mean ± SD of 5 determinations. The relative migration distance was normalized to the average value of scrambled group

To assess the effect of ROR2 receptors on Wnt5a-induced osteosarcoma cell migration, we generated the stable ROR2 knockdown MG-63 cells and transfected U2OS cells with specific siRNA targeting ROR2 and measured the cell migration by wound healing assays. The shRNA or siRNA against human ROR2 knocked down ROR2 expression by approximately 50% as assessed by Western blotting in MG-63 and U2OS cells (Fig. 1a), which resulted in a significant reduction of Wnt5a-induced cell migration (Fig. 1b and c). Thus, ROR2 participates in Wnt5a-induced osteosarcoma cell migration.

### ROR2 is essential for Wnt5a-induced RhoA activity

**Fig. 2:**
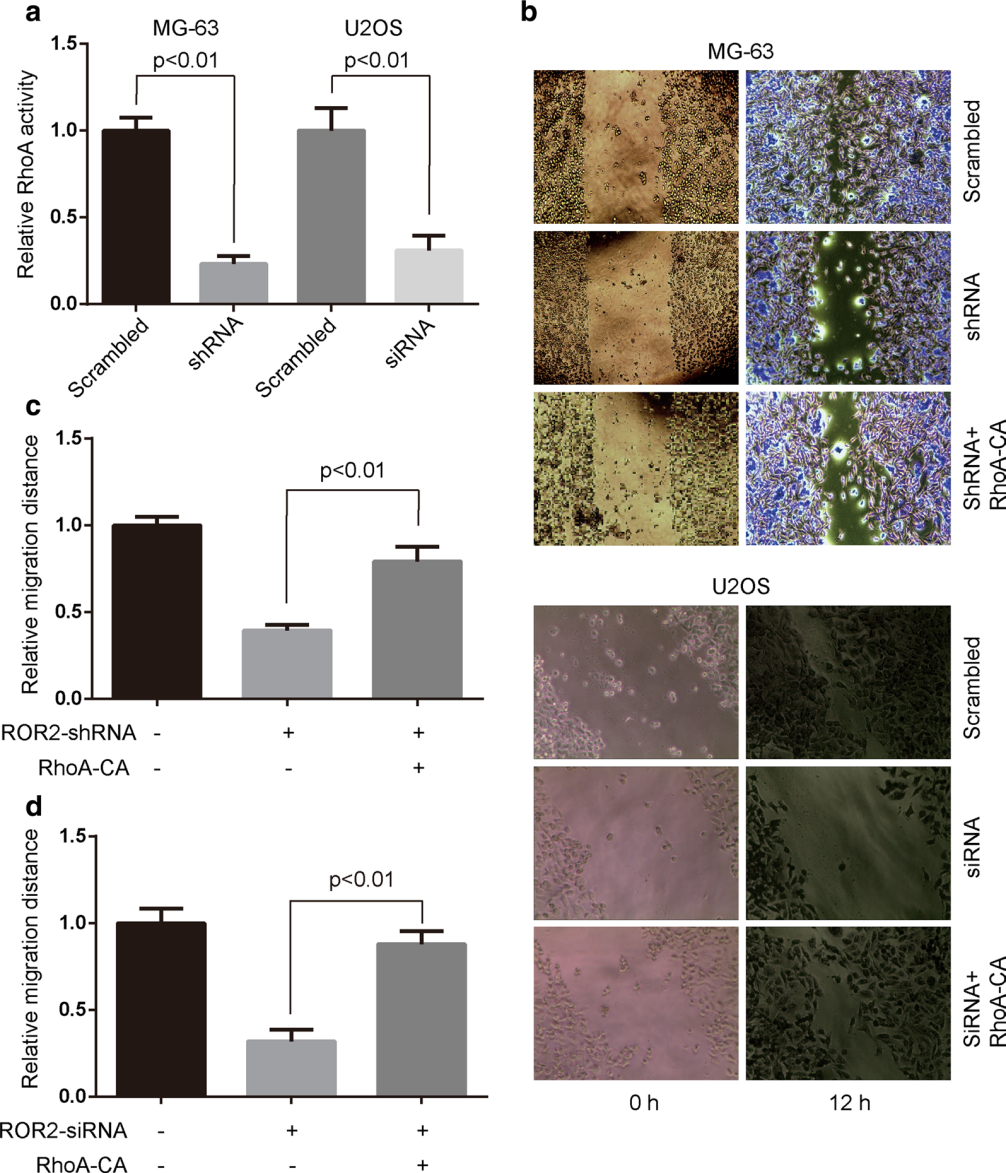
Knockdown of ROR2 decreases Wnt5a-induced RhoA activity and cell migration. **a** Stable ROR2 knockdown MG-63 cells and osteosarcoma U2OS cells transfected with ROR2-siRNA or scrambled siRNA were incubated with 100 ng/mL Wnt5a and harvested at 30 min after the start of Wnt5a treatment. The determination of RhoA activity was described in “Methods”. Data were presented as mean ± SD of 5 determinations. The relative RhoA activity was normalized to the average value of scrambled group. **b**, **c**, and **d** Stable ROR2 knockdown MG-63 cells (**b**, **c**) and osteosarcoma U2OS cells (**b**, **d**) transfected with ROR2-siRNA and/or RhoA-V14 (RhoA-CA), were subjected to wound healing assays. Cells incubated with 100 ng/mL Wnt5a were allowed to migrate for 12 h. Data were presented as mean ± SD of 5 determinations. The relative migration distance was normalized to the average value of the control group

**Fig. 3:**
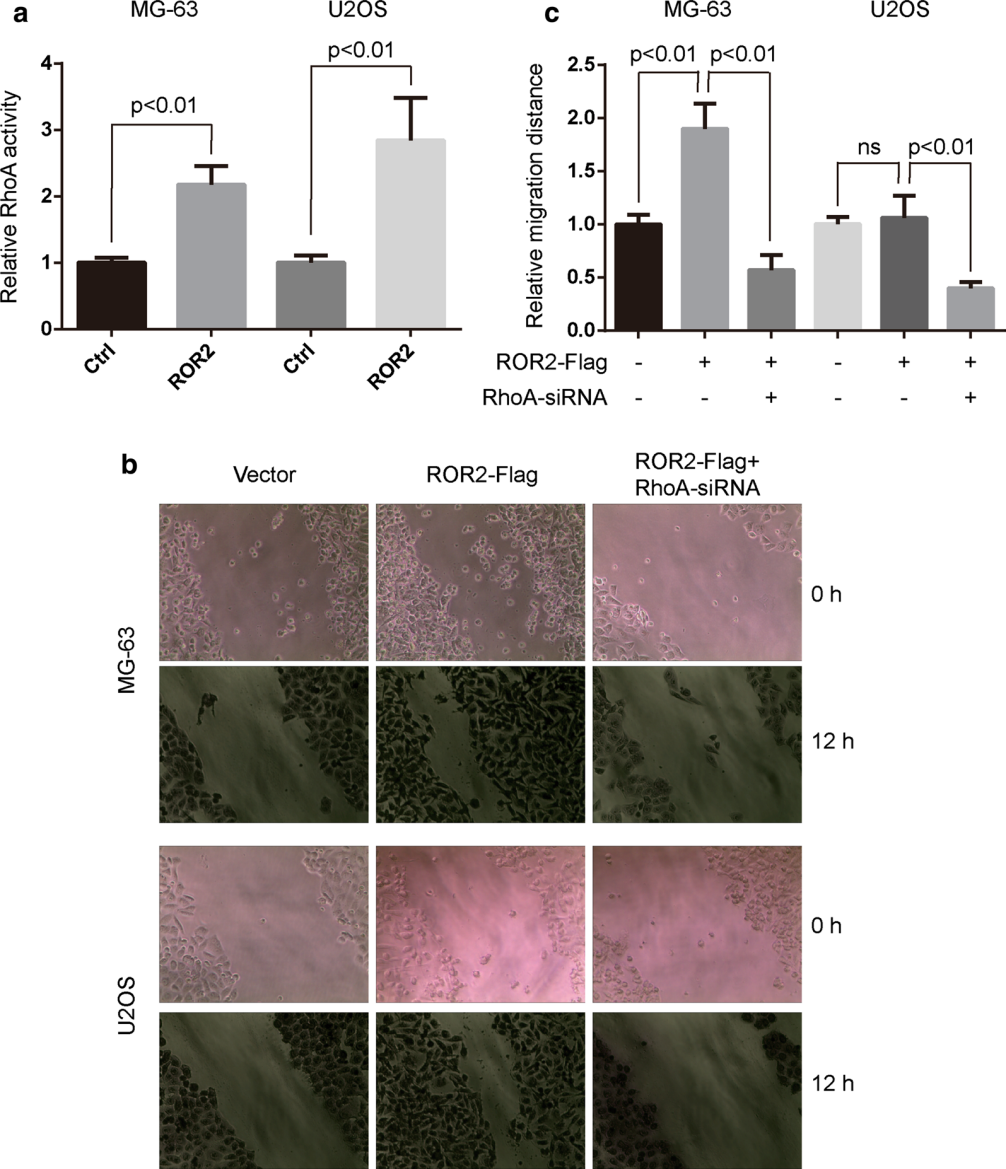
Overexpression of ROR2 increases Wnt5a-induced RhoA activity and cell migration. **a** Human osteosarcoma MG-63 and U2OS cells, transfected with ROR2-Flag or vectors (Ctrl), were incubated with 100 ng/mL Wnt5a and harvested at 30 min after the start of Wnt5a treatment. The determination of RhoA activity was described in “Methods”. Data were presented as mean ± SD of 5 determinations. The relative RhoA activity was normalized to the average value of scrambled group. **b** and **c** MG-63 and U2OS cells, transfected with ROR2-Flag and/or RhoA-siRNA, were subjected to wound healing assays. Cells incubated with 100 ng/mL Wnt5a were allowed to migrate for 12 h. Data were presented as mean ± SD of 5 determinations. The relative migration distance was normalized to the average value of the control group. *Ns* no significance

The finding that Wnt5a elevates RhoA activation in MG-63 cells [6] prompted us to determine whether ROR2 receptor was required for Wnt5a-induced RhoA activity. Wnt5a-induced RhoA activity was largely abolished by shRNA or siRNA specific against ROR2 (Fig. 2a), and significantly increased by ROR2 overexpression in MG-63 and U2OS cells (Fig. 3a). These results suggest that ROR2 is required for Wnt5a-induced RhoA activity of MG-63 and U2OS cells.

Next, we used constitute activity constructs (RhoA-CA, RhoA-V14) to elevate RhoA activity in osteosarcoma cells and checked whether the reductive migration rate by ROR2 knockdown could be rescued. RhoA-CA (RhoA-V14) was capable of increasing the cell migration in ROR2-knockdown MG-63 and U2OS cells (Fig. 2b, c and d). Moreover, siRNA specific against RhoA retarded Wnt5a/ROR2-mediated cell migration of MG-63 and U2OS cells (Fig. 3b and c). These findings suggest that ROR2/RhoA signaling mediates the Wnt5a-induced cell migration of osteosarcoma cells.

### PI3Kα/Akt signaling acts as the downstream of Wnt5a/ROR2

**Fig. 4:**
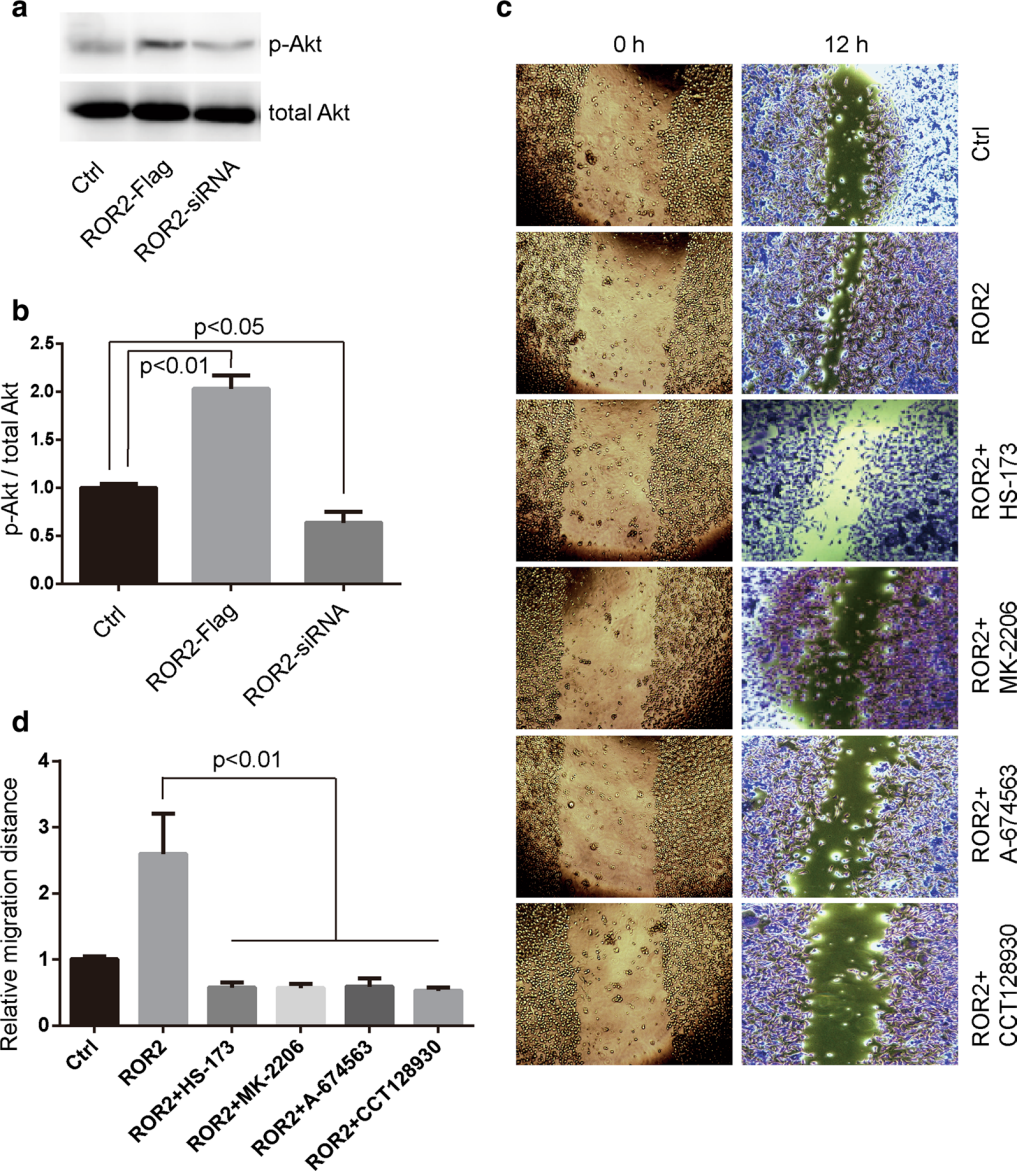
PI3Kα and Akt acts as the downstream of Wnt5a/ROR2 signaling. **a** and **b** Human osteosarcoma MG-63 cells, transfected with ROR2-Flag or ROR2-siRNA, were incubated with 100 ng/mL Wnt5a and harvested at 15 min after the start of Wnt5a treatment. The expression and activity of Akt were determined by Western blotting assays. Data were presented as mean ± SD of 3 determinations. The ratio of p-Akt and total Akt was normalized to the average value of control group. **c** and **d** MG-63 cells, transfected with ROR2-Flag or vectors, were treated with 1 nmol/L HS-173 (PI3Kα inhibitor), 10 nmol/L MK-2206 (Akt inhibitor), 10 nmol/L A-674563 (Akt1 inhibitor), or 10 nmol/L CCT128930 (Akt2 inhibitor) for 1 h, then subjected to wound healing assays. Cells incubated with 100 ng/mL Wnt5a were allowed to migrate for 12 h. Data were presented as mean ± SD of 5 determinations. The relative migration distance was normalized to the average value of control group

Given that PI3Kα/Akt signaling (specific PI3Kα, Akt1 and Akt2 isoforms) mediate Wnt5a-induced the migration of osteosarcoma cells [5, 6], we propose that PI3Kα/Akt act as the downstream of Wnt5a and ROR2. MG-63 cells, transfected with ROR2-Flag or ROR2-siRNA, were treated with 100 ng/mL of Wnt5a. The cells were harvested at 15 min after the start of Wnt5a treatment, followed by SDS-PAGE and Western blotting analyses (Fig. 4a and b). Akt showed the significantly increased or decreased signs of phosphorylation at Ser473 after ROR2-Flag or ROR2-siRNA transfection, respectively (Fig. 4a and b).

Moreover, we want to know whether inhibitors of PI3Kα/Akt signaling block Wnt5a/ROR2-mediated cell migration. MG-63 cells, transfected with ROR2-Flag, were pretreated with 1 nmol/L HS-173 (PI3Kα inhibitor), 10 nmol/L MK-2206 (Akt inhibitor), 10 nmol/L A-674563 (Akt1 inhibitor), or 10 nmol/L CCT128930 (Akt2 inhibitor), respectively, then were incubated with 100 ng/mL Wnt5a. The Wnt5a/ROR2-mediated cell migration was largely blocked by pretreatment of HS-173, MK-2206, A-674563 and CCT128930 in MG-63 cells, respectively (Fig. 4c and d). These data indicate that PI3Kα/Akt signaling acts as the downstream of Wnt5a/ROR2 and regulates the migration of osteosarcoma cells.

### Wnt5a/ROR2 signaling does not alter osteosarcoma cell proliferation

**Fig. 5:**
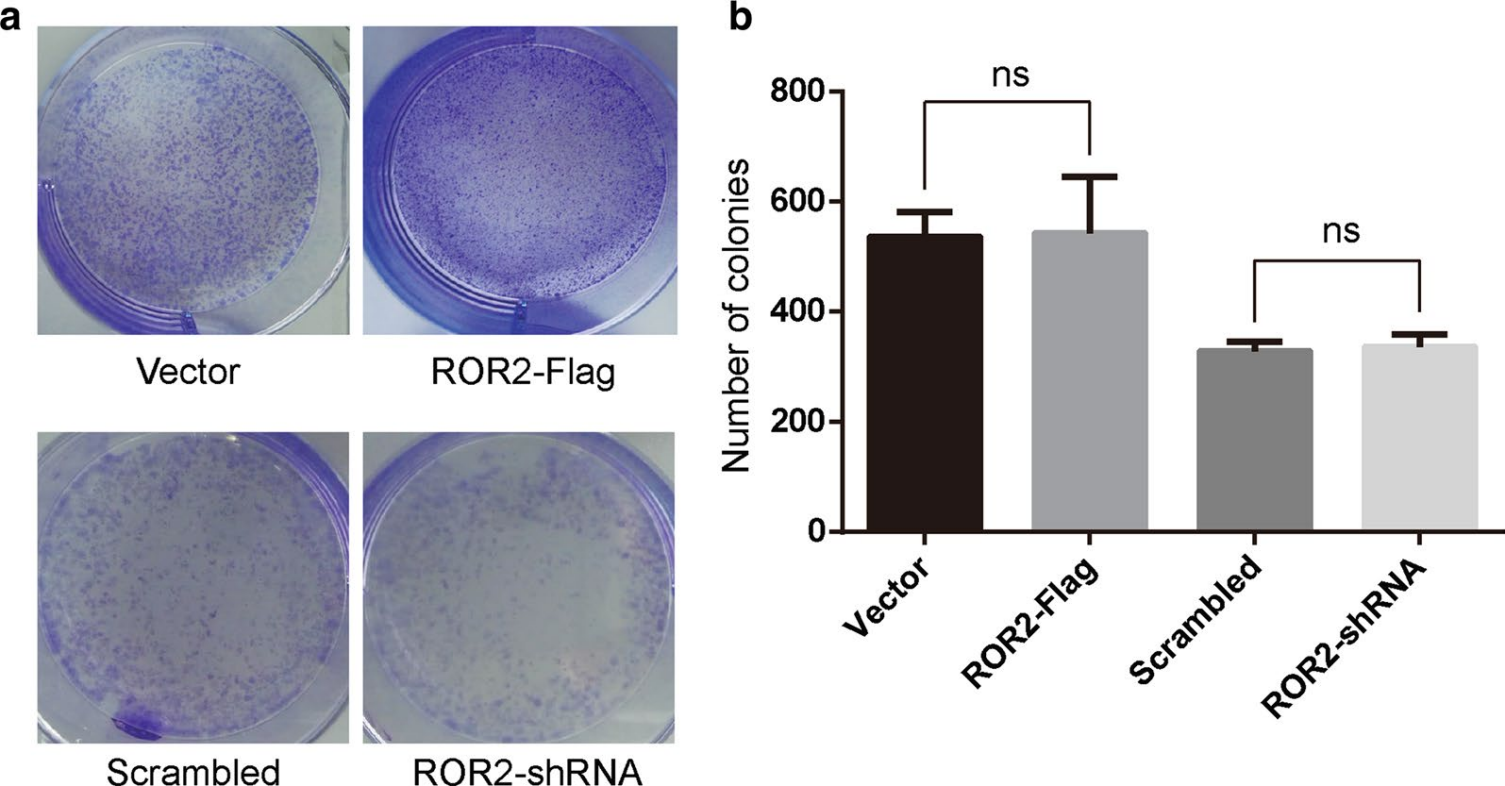
Wnt5a/ROR2 signaling does not alter osteosarcoma cell proliferation. **a** Human osteosarcoma MG-63 cells transfected with ROR2-Flag or stable ROR2 knockdown MG-63 cells were treated with 100 ng/mL of Wnt5a and subjected to the clonogenic assay. **b** The number of colonies were counted under a microscope. Data were presented as mean ± SD of 3 determinations. *Ns* no significance

Wnt5a/ROR2 signaling is associated with suppression of β-catenin/TCF-dependent transcriptional activity and down-regulated the expression of cyclin D1 in erythroleukemia cells [17], suggesting its anti-tumor role on cell proliferation. Here, we transfected osteosarcoma MG-63 cells with ROR2-Flag or stable ROR2 knockdown MG-63 cells, then were incubated with 100 ng/mL of Wnt5a and subjected to clonogenic assays. Neither overexpression nor knockdown of ROR2 did not alter the proliferation of osteosarcoma cells (Fig. 5a and b). In conclusion, ROR2 receptor, acting as the upstream of PI3Kα/Akt/RhoA signaling, is required for Wnt5a-induced the migration, not the proliferation of osteosarcoma cells.

## Discussion

Receptor tyrosine kinase-like orphan receptor is a receptor family consisting of two closely related type I transmembrane proteins ROR1 and ROR2. Owing to mutations in their canonical motifs required for proper kinase activity, RORs are classified as pseudokinases lacking detectable catalytic activity [18]. ROR2 is up-regulated in a lot of human tumors including osteosarcoma, melanoma, renal cell carcinoma, prostate carcinoma, colorectal cancer, squamous cell carcinomas of the head and neck, stromal tumors, and breast cancers [19–26]. Wnt5a is a prototypic ligand which activates a β-catenin independent pathway in Wnt signaling [27, 28]. Owing to the synchronous highly expression pattern of Wnt5a in breast cancer, gastric cancer, non-small-cell lung cancer, prostate cancer [22, 29–32], we predict that Wnt5a and ROR2 may act as co-effector in certain specific tumors. Wnt5a/ROR2 signaling elevates expression and secretion of CXCL16 in mesenchymal stem cells (MSCs), leading to the promotion of its proliferation [30]. Here, we demonstrate that ROR2 mediates Wnt5a-induced cell migration of osteosarcoma.

Our previous study finds that Wnt5a mediates the migration of osteosarcoma cells via elevating the PI3K/Akt and RhoA signaling [5, 6]. Down-regulation of PI3K/Akt/GSK3β signaling in gastric cancer cells suppresses Wnt5a-induced activation of RhoA and cell migration [33]. In this study, overexpression of constitute active RhoA rescues Wnt5a-induced cell migration blocked by shRNA or siRNA against ROR2 in osteosarcoma cells. Specific inhibitors targeting PI3K and Akt retard Wnt5a-induced cell migration in ROR2-overexpressed osteosarcoma cells. These results indicates that the potential role of Wnt5a/ROR2/PI3K/Akt/RhoA signaling is an accelerator in osteosarcoma metastatic behavior.

Wnt5a and its receptor ROR2 act synergistically to increase autocrine signaling and inhibits canonical Wnt signaling in myeloid leukemia cells [17]. A large number of studies demonstrate that canonical Wnt signaling facilitates the proliferation in both embryo development and tumor progression [34–38]. Finally, we find that Wnt5a/ROR2 signaling does not affect the proliferation of osteosarcoma cells.

## Conclusions

We present the evidence here that ROR2 mediates Wnt5a-induced osteosarcoma cell migration via PI3K/Akt and RhoA signaling. These findings elucidate a molecular pathway linking ROR2 signaling to Wnt5a ligand in cell motility. This result will contribute to further understanding of biological roles of Wnt5a/ROR2/PI3K/Akt/RhoA in cell migration of osteosarcoma and other cancers.

## Abbreviations

PI3K: phosphatidylinositol-3 kinase
Fzd: frizzled family receptors
LRP: low-density lipoprotein receptor-related protein
ROR: receptor tyrosine kinase-like orphan receptor
MSC: mesenchymal stem cell
DMEM: Dulbecco-modified Eagle’s medium
FBS: fetal bovine serum
